# Integrating radiomics into predictive models for low nuclear grade DCIS using machine learning

**DOI:** 10.1038/s41598-025-92080-y

**Published:** 2025-03-03

**Authors:** Yimin Wu, Daojing Xu, Zongyu Zha, Li Gu, Jieqing Chen, Jiagui Fang, Ziyang Dou, Pingyang Zhang, Chaoxue Zhang, Junli Wang

**Affiliations:** 1 Department of Ultrasound, WuHu Hospital, East China Normal University (The Second People’s Hospital, WuHu), No.6 Duchun Road, Jinghu District, Wuhu, 241000 Anhui China; 2 Department of Echocardiography, Nanjing First Hospital, Nanjing Medical University, Changle Road 68, Nanjing, 210006 Jiangsu China; 3 Department of Ultrasound, The First Affiliated Hospital of Anhui Medical University, No.218 Jixi Road, Shushan District, Hefei, 230022 Anhui China

**Keywords:** Ductal carcinoma in situ, Machine learning, Radiomic, Ultrasound, Mammography, Cancer, Medical research

## Abstract

Predicting low nuclear grade DCIS before surgery can improve treatment choices and patient care, thereby reducing unnecessary treatment. Due to the high heterogeneity of DCIS and the limitations of biopsies in fully characterizing tumors, current diagnostic methods relying on invasive biopsies face challenges. Here, we developed an ensemble machine learning model to assist in the preoperative diagnosis of low nuclear grade DCIS. We integrated preoperative clinical data, ultrasound images, mammography images, and Radiomic scores from 241 DCIS cases. The ensemble model, based on Elastic Net, Generalized Linear Models with Boosting (glmboost), and Ranger, improved the ability to predict low nuclear grade DCIS preoperatively, achieving an AUC of 0.92 on the validation set, outperforming the model using clinical data alone. The comprehensive model also demonstrated notable enhancements in integrated discrimination improvement and net reclassification improvement (*p* < 0.001). Furthermore, the Radiomic ensemble model effectively stratified DCIS patients by risk based on disease-free survival. Our findings emphasize the importance of integrating Radiomic into DCIS prediction models, offering fresh perspectives for personalized treatment and clinical management of DCIS.

## Introduction

Ductal Carcinoma In Situ (DCIS) accounts for more than a quarter of all newly diagnosed breast cancer cases. With the widespread application of breast cancer screening, its detection rate is on the rise^1^. The typical approach for treating DCIS involves adjuvant therapy after surgery, which may consist of radiation therapy, endocrine therapy, or an integration of both^2^. The issue of overdiagnosis of DCIS, which is not an inevitable precursor to invasive breast cancer, has recently attracted widespread attention^3,4^. Most lesions of DCIS do not develop into invasive illnesses and are unlikely to cause symptoms or lead to death^5,6^. Certain clinicopathologic variables encompass younger age, greater lesion size, hormone receptor-negative, Human Epidermal growth factor Receptor 2 (HER2) positivity, high nuclear grade (HNG), comedo necrosis, and positive margin status are linked to death or recurrence in DCIS patients^7,8^, the significance and connection between these characteristics are not well understood, which complicates the precise prediction of which DCIS patients are most likely to develop invasive cancer.

High nuclear grade ductal carcinoma in situ (HNG DCIS) is linked to high-grade invasive carcinoma and exhibits more pronounced biological aggressiveness compared to Low nuclear grade ductal carcinoma in situ (LNG DCIS)^9,10^. Prior research examining the clinical advantages of surgically removing LNG DCIS revealed comparable survival rates in women with LNG DCIS^3,11^, regardless of whether they underwent surgery. Given that nuclear grade has been shown to be a predictive factor for unfavorable results^12,13^, current prospective randomized trials, such as those conducted by Francis and Hwang, focus on monitoring patients with low to intermediate nuclear grade DCIS without surgical intervention^14,15^. Identifying LNG DCIS before surgery is essential for these individuals to ensure proper care of DCIS. However, preoperatively predicting LNG DCIS is difficult because of the great heterogeneity of DCIS and the inherent constraints of biopsy samples, often leading to underestimation or missed detection of invasive lesions^16,17^. Hence, there is a pressing need to create a novel technique for predicting LNG DCIS before surgery.

Magnetic resonance imaging (MRI) is widely regarded as the most effective imaging technique for identifying DCIS. A study comparing MRI and mammography found that MRI has a sensitivity of 79.3% for pure DCIS, while mammography has a sensitivity of 69%, with no statistically significant difference (*P* = 0.345)^18^. However, the high cost, time-consuming nature, and complexity of MRI procedures limit its widespread use in DCIS screening. Mammography (MG) and ultrasound (US) are commonly used in clinical settings for the detection, diagnosis, and monitoring of DCIS, unlike MRI. MG is particularly sensitive to microcalcifications, with approximately 80% of DCIS presenting as asymptomatic calcifications on MG^19^. Microcalcifications are more common in HNG DCIS and DCIS with necrosis, often appearing as fine, pleomorphic, or branching calcifications, whereas round calcifications are associated with LNG DCIS^20^. Nevertheless, MG can result in false negatives, particularly in patients with dense breast tissue, absence of microcalcifications, or small lesions. US has an advantage in detecting non-calcified DCIS lesions in dense breast tissue^21^. Prior research has indicated that additional ultrasound screening in females with dense breast tissue can identify small, otherwise hidden breast tumors^22^. For non-calcified DCIS, US has a detection rate of 95% compared to 68% for MG, typically manifesting as irregular, hypoechoic, vascularized masses with parallel orientation and no posterior features^19,22^. However, these standard imaging descriptors alone are insufficient to differentiate the nuclear grade of DCIS. Additionally, age and symptoms are considered significant clinical factors influencing DCIS outcomes^17,23^.

Radiomic is a new type of medical imaging analysis that uses various data mining algorithms and statistical tools to examine quantitative imaging characteristics, offering predictive or prognostic insights. Radiomic has successfully evaluated and predicted various challenging clinical tasks by utilizing feature engineering to develop appropriate machine learning models^24–27^. For predicting LNG DCIS, Zhu et al.^28^ developed a deep learning Radiomic model based on US, achieving an AUC of 0.63. Chou et al.^29^ demonstrated that MRI-based radiomic features approached statistical significance in distinguishing high from non-high-grade DCIS. Nevertheless, there is a scarcity of research that integrates ultrasound and mammography to forecast the likelihood of developing low-grade non-invasive ductal carcinoma in situ. We hypothesize that US and MG can complement each other in the Radiomic domain to achieve better predictive performance. Hence, in this study, we developed an integrated machine learning model that combines multimodal radiomic features derived from ultrasound (US ) and mammography (MG ) with clinical data to preoperatively predict LNG DCIS. Our results indicate that this model achieved excellent predictive performance in the independent validation cohort (AUC = 0.92), and by significantly improving both IDI and NRI (*p* < 0.001), further enhanced its diagnostic utility. Additionally, the model effectively stratified patients according to their disease-free survival (DFS) risk, thereby offering valuable guidance to clinicians in identifying high-risk patients before surgery, optimizing treatment strategies, and reducing unnecessary invasive examinations. Through the incorporation of interpretive analytical tools such as SHAP, the decision-making process of our model is rendered more transparent, providing a solid foundation for future multicenter validation and broader clinical adoption in individualized and precision-based DCIS management.

## Materials and methods

### Patient data collection

**Fig. 1:**
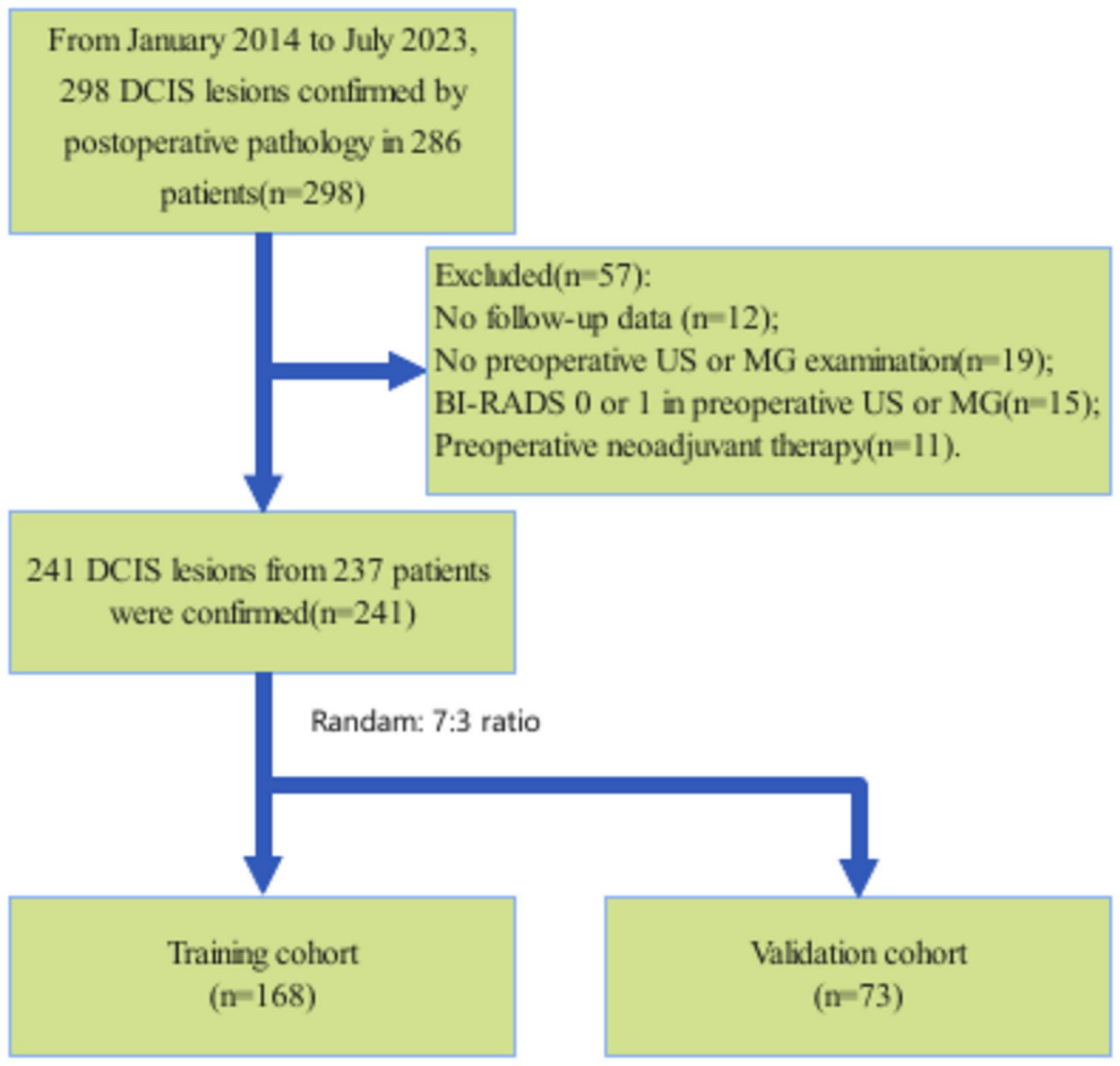
The patient inclusion flowchart.

The research was conducted in accordance with the Declaration of Helsinki and was approved by the Ethics Committee of the Second People’s Hospital of Wuhu City, which waived the need for written informed consent due to the study’s retrospective nature. We collected data from DCIS patients who were pathologically confirmed through surgery at our institution between January 2014 and July 2023. The inclusion criteria for this study were as follows: (i) patients pathologically diagnosed with DCIS; (ii) availability of preoperative ultrasound (US) and mammography (MG) examinations; and (iii) available follow-up data. The exclusion criteria included: (i) patients with no preoperative US or MG examination; (ii) BI-RADS 0 (requiring further imaging evaluation) or BI-RADS 1 (negative) in preoperative US or MG; and (iii) preoperative neoadjuvant therapy. Data on age, symptoms, nuclear grade, estrogen receptor (ER) status, progesterone receptor (PR) status, HER2 status, and Ki-67 index were obtained from electronic health records for all patients. Post-surgery pathology was used as the benchmark, dividing cases into two categories: low nuclear grade DCIS and DCIS with intermediate to high nuclear grade.ER and PR positivity were determined by the presence of nuclear staining in over 1% of cells when viewed under a × 10 magnification. HER2 positivity was determined by a staining score of 3 + or gene amplification positivity. If the HER2 score was 2 + and gene amplification results were not available, HER2 status was deemed uncertain. Ki-67 positivity was determined by the presence of nuclear staining in over 14% of cancerous cells. While BI-RADS 3 lesions are typically recommended for follow-up imaging rather than biopsy or surgery, in this study, cases with BI-RADS 3 lesions were included if pathological confirmation was obtained. This approach accounts for real-world clinical scenarios where biopsy or surgery might be performed due to factors such as lesion growth, imaging discordance between US and MG, or patient-specific concerns. Follow-up information was retrospectively collected from electronic health records to evaluate disease-free survival (DFS). DFS was defined as the time from surgery to the first recurrence, progression, or death, whichever occurred first. Cases without events were censored at the last recorded follow-up. The dataset was randomly divided into a training cohort and a validation cohort in a 7:3 ratio for each category. The patient inclusion flowchart is shown in Fig. 1.

### US and MG imaging collection

All patients underwent preoperative ultrasound (US) and mammography (MG) examinations. Ultrasound examinations were conducted by five experienced radiologists, each with over ten years of experience in breast ultrasound imaging. The ultrasound machines utilized were Siemens Auson S2000, Logic E9, Philips EPIQ 5, Siemens Sequoia, and Philips EPIQ 7, all equipped with linear array transducers with frequencies between 6–18 MHz, 7–12 MHz, 5–14 MHz, and 5–12 MHz, respectively. Two radiologists, each with 12 years of experience in diagnosing breast ultrasound, reviewed gray-scale ultrasound images with lesions in a retrospective manner. When there was a difference of opinion, a senior radiologist with two decades of experience in breast ultrasound imaging made the ultimate decision. The largest diameter of the lesion (in millimeters), lesion type (mass or non-mass lesion), and the US-BI-RADS classification were recorded. The Senographe Digital mammography system (GE Healthcare, Milwaukee, WI, USA) was utilized for the mammography procedure. Standard CC and MLO images were captured, with extra images taken as needed. Retrospectively collected data from mammography reports included identification of microcalcifications and classification according to MG-BI-RADS criteria.

### Image segmentation and radiomic feature extraction

Two radiologists, each with 12 years of experience in breast ultrasound diagnosis, independently reviewed US and MG images in the research. They did not have access to the histological nuclear grading information of DCIS, but they knew that these patients were eventually diagnosed with DCIS. All segmentation tasks were performed by the first radiologist using the Segment Editor module integrated in 3D Slicer (version 5.2.1; http://www.slicer.org), and consensus was reached with the second radiologist. Prior to segmentation, images were cropped and resampled to a pixel size of 1 × 1 mm^2^ to remove any redundant information and to maintain consistent spatial resolution. Given the heterogeneity of DCIS images, various strategies were employed for segmentation. For microcalcifications, the ‘Threshold’ tool was initially used to automatically detect the range of microcalcifications by setting a high threshold value, effectively identifying microcalcified areas in the images. This was followed by manual adjustments using the ‘Paint’ and ‘Erase’ functions to ensure accurate identification of all microcalcified regions and to exclude potential artifacts. For non-mass lesions, the ‘Level tracing’ tool was used to initially outline the boundaries, with manual adjustments using ‘Paint’ and ‘Erase’ functions to ensure accurate segmentation of the lesions. Massive growths were outlined manually using the ‘Paint’ and ‘Draw’ tools to define the region of interest (ROI) around the growths, while also making adjustments with the ‘Erase’ feature. PyRadiomic was utilized to extract quantitative radiomic characteristics from the regions of interest (ROIs). These features included shape, texture, and intensity histogram data to capture the multidimensional characteristics of the lesions. To assess the reproducibility of radiomic features under varying observational conditions, we employed the Intraclass Correlation Coefficient (ICC) to evaluate both intra-observer (the same radiologist at different time points) and inter-observer (different radiologists) reliability. Higher ICC values indicate greater robustness of the extracted features against observer-related variability. After the initial segmentation, we randomly selected 30 DCIS cases and repeated the segmentation two weeks later to minimize recall bias. This sample size is consistent with recommended guidelines and previous literature, which generally suggest including at least 30 cases to reliably estimate ICC values^30^^.^ Features with ICC values above 0.8 were considered to demonstrate excellent reliability and were retained for subsequent analyses.

### Radiomic feature selection

We employed a two-step process to select robust radiomic features exclusively within the training subset, ensuring that the test subset was not involved at any stage of feature selection to prevent data leakage. Initially, radiomic features with an ICC exceeding 0.8 were selected and normalized through z-score standardization. Next, we developed the US, MG, and US + MG Radiomic models using LASSO regression with tenfold cross-validation and evaluated the model performance using the receiver operating characteristic curve (ROC). Subsequently, the radiomic score (radscore) was calculated based on the final selected features and their weight coefficients from the models, for subsequent ensemble model training and evaluation. This approach was chosen because it enables the identification of the most predictive radiomic features while reducing dimensionality and avoiding overfitting^31,32^.

### Development of the radiomic ensemble model

**Fig. 2:**
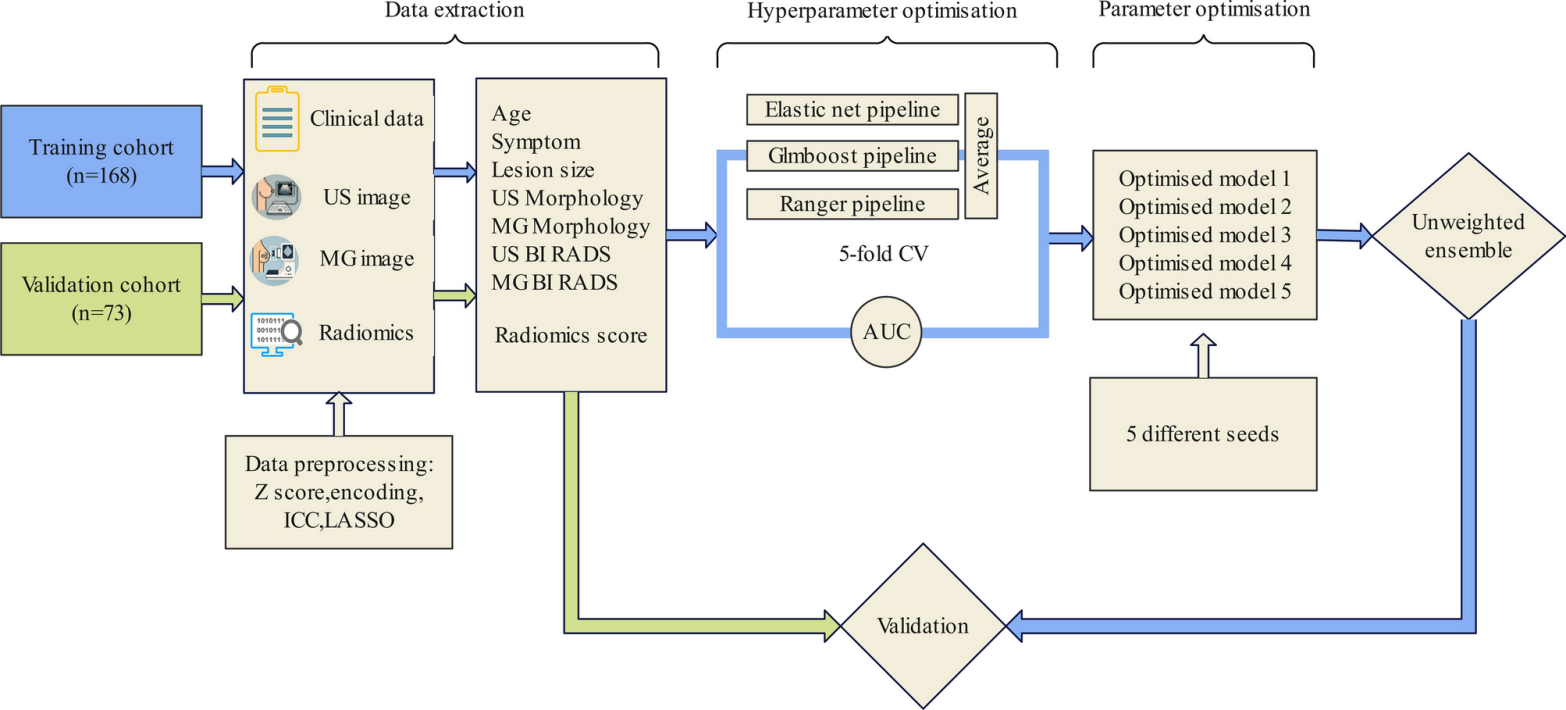
Illustration of the workflow.

We developed an integrated machine learning framework to predict LNG DCIS, incorporating all available clinical, imaging, and Radiomic features (Supplementary Table S1). Prior to modeling, clinical and imaging features were preprocessed: continuous variables (e.g., age) were standardized using z-score, while categorical variables (e.g., symptoms) were encoded using treatment coding. Variance inflation factor (VIF) values were calculated to identify and remove highly collinear features. The model was trained and optimized using the training cohort and subsequently frozen for evaluation on the validation cohort. We constructed two models based on feature availability: a clinical model, including age, symptoms, and five imaging features, and a Radiomic ensemble model, which integrated radiomic score with the clinical model features. The clinical model, based solely on preoperative clinical and imaging features, served as a baseline to evaluate the incremental predictive value provided by the radiomic score in the ensemble model. Predictions for both models were generated using Elastic Net, Generalized Linear Models with Boosting (glmboost), and Ranger, individually and as part of an unweighted ensemble classifier that integrated the predictions of these algorithms. All algorithms were implemented using the mlr3 package in R, embedded in dedicated mlr3 pipelines. To maximize the AUC, we employed a random search strategy to optimize hyperparameters during five-fold cross-validation, as detailed in Supplementary Table S2. For robustness, the procedure was repeated five times using distinct random seeds. The final ensemble prediction was obtained by averaging the results of five independent predictions. Each prediction was generated using the ensemble model architecture, combining Elastic Net, glmboost, and Ranger, with variations introduced by different random seeds during cross-validation to improve robustness and reduce overfitting. The workflow of model development and evaluation, including feature preprocessing, classifier integration, and ensemble prediction generation, is illustrated in Fig. 2.

### Incremental predictive value of the radiomic ensemble model

We evaluated the additional predictive power of the Radiomic ensemble model compared to the clinical model through integrated discrimination improvement (IDI) and net reclassification improvement (NRI). ROC curve analysis was used to evaluate different measurements, such as AUC, accuracy, sensitivity, specificity, NPV, and PPV. The clinical effectiveness of each model was compared using decision curve analysis (DCA).

### Predictive potential for DCIS recurrence

We used the Youden index to assess the model’s ability to predict the recurrence of DCIS by establishing cutoff values and dividing patients into low-risk and high-risk groups based on whether they were above or below the cutoff. Kaplan–Meier analysis was conducted to assess the significance of variations in disease-free survival (DFS) between the low-risk and high-risk subgroups using log-rank tests.

### Model interpretation and feature importance

To interpret the model’s output and evaluate feature importance, we employed Shapley Additive Explanations (SHAP) values. SHAP values, based on game theory, measure how much each feature influences the model’s predictions. We used SHAP values to calculate the importance of each feature in the training data, creating plots that show how significant each feature is in the model’s predictions. For specific instance explanations, SHAP values were calculated for individual samples to provide detailed feature contribution analyses, aiding in understanding the model’s decision-making process.

### Statistical analysis

R (version 4.4.0) was utilized for conducting statistical analyses. A *p*-value less than 0.05 (for a two-sided test) was deemed to be statistically significant. Continuous data were analyzed using t-tests or non-parametric Mann–Whitney U tests. Either Pearson’s chi-square test or Fisher’s exact test was utilized to assess disparities in categorical factors. The evaluation of model performance included the calculation of the area under the ROC curve (AUC) as well as various other performance metrics such as accuracy, sensitivity, specificity, PPV, and NPV. Disease-free survival was estimated using the Kaplan–Meier technique and log-rank tests. We utilized the pROC package (version 1.18.5; https://cran.r-project.org/web/packages/pROC/index.html) in R to generate ROC graphs and determine the AUC. Bootstrap methods were employed to compute confidence intervals. The “Hmisc” package was used to calculate IDI and NRI, while the “dcurves” package was used to perform DCA.

## Results

### Baseline characteristics of patients

This study included 237 patients with 241 cases of DCIS, 4 of which were bilateral breast DCIS cases, based on the exclusion criteria. The median age of the patients was 52 years (IQR, 42–61 years), with 21.2% presenting with palpable lumps and 9.1% with nipple discharge. The 241 cases were randomly assigned to a training cohort (n = 168/241, 69.7%) and a validation cohort (n = 73/241, 30.3%).Within the group being trained, there were 45 instances of LNG DCIS, accounting for 26.8%, and 123 instances of intermediate to high nuclear grade DCIS, making up 73.2%; in the group being validated, there were 19 instances of LNG DCIS, representing 26.0%, and 54 instances of intermediate to high nuclear grade DCIS, accounting for 74.0%.Table 1 outlines the initial traits of patients categorized by surgical pathology. The distributions between the two cohorts were balanced (*p*-value range, 0.109–1). Table 2 shows the distribution of study samples in the trial. Significant variations were observed between the LNG DCIS and intermediate to high nuclear grade DCIS groups in terms of US and MG morphology, US-BI-RADS, MG-BI-RADS, ER, PR, HER2, and Ki-67 levels (*p* < 0.05). In the validation cohort, PR was the sole variable that did not exhibit any notable discrepancies. There were no significant variations in age, symptoms, or nodule size among the groups.

**Table 1:** Distribution of baseline characteristics based on surgical pathology.

	**[ALL]**	**Training cohort**	**Validation cohort**	***p***
	***N* = *241***	***N* = *168***	***N* = *73***	
Age	52.0 [46.0;61.0]	52.0 [45.0;59.2]	53.0 [47.0;62.0]	0.273
Symptom:				0.840
None	168 (69.7%)	119 (70.8%)	49 (67.1%)	
Nipple discharge	22 (9.13%)	15 (8.93%)	7 (9.59%)	
Palpable lump	51 (21.2%)	34 (20.2%)	17 (23.3%)	
Lesion size	23.0 [16.0;33.0]	23.0 [15.0;31.2]	26.0 [19.0;35.0]	0.109
US Morphology:				0.199
Mass	112 (46.5%)	73 (43.5%)	39 (53.4%)	
Nonmass lesion	129 (53.5%)	95 (56.5%)	34 (46.6%)	
MG Morphology:				0.895
Noncalcified	86 (35.7%)	59 (35.1%)	27 (37.0%)	
Calcified	155 (64.3%)	109 (64.9%)	46 (63.0%)	
US-BI RADS:				0.938
3	37 (15.4%)	23 (13.7%)	13(17.8%)	
4A	37 (15.4%)	26 (15.5%)	11 (15.1%)	
4B	65 (27.0%)	47 (28.0%)	18 (24.7%)	
4C	77 (32.0%)	54 (32.1%)	23 (31.5%)	
5	25 (10.4%)	18 (10.7%)	8 (11.0%)	
MG-BI RADS:				0.706
2	17 (7.05%)	9 (5.36%)	6 (8.22%)	
3	45 (18.7%)	33 (19.6%)	11 (15.1%)	
4A	50 (20.7%)	32 (19.0%)	18 (24.7%)	
4B	81 (33.6%)	61 (36.3%)	22 (30.1%)	
4C	44 (18.3%)	30 (17.9%)	15 (20.5%)	
5	4 (1.66%)	3 (1.79%)	1 (1.37%)	
Nuclear grade:				1.000
Intermediate to high nuclear grade	177 (73.4%)	123 (73.2%)	54 (74.0%)	
Low nuclear grade	64 (26.6%)	45 (26.8%)	19 (26.0%)	
ER:				0.595
Negative	59 (24.5%)	39 (23.2%)	20 (27.4%)	
Positive	182 (75.5%)	129 (76.8%)	53 (72.6%)	
PR:				0.526
Negative	58 (24.1%)	38 (22.6%)	20 (27.4%)	
Positive	183 (75.9%)	130 (77.4%)	53 (72.6%)	
HER2:				0.442
NA	22 (9.13%)	13 (7.74%)	9 (12.3%)	
Negative	160 (66.4%)	115 (68.5%)	45 (61.6%)	
Positive	59 (24.5%)	40 (23.8%)	19 (26.0%)	
KI67:				0.880
Negative	188 (78.0%)	132 (78.6%)	56 (76.7%)	
Positive	53 (22.0%)	36 (21.4%)	17 (23.3%)	

DCIS, ductal carcinoma in situ; ER, estrogen receptor; PR, progesterone receptor; HER2, human epidermal growth factor receptor 2; MG, mammography; US, ultrasound; BI-RADS, Breast Imaging Reporting and Data System.

**Table 2:** The distribution of research samples in the experiment.

	Training cohort				Validation cohort			
	[ALL]	Intermediate to high nuclear grade	Low nuclear grade	*p*	[ALL]	Intermediate to high nuclear grade	Low nuclear grade	*p*
	*N* = *168*	*N* = *123*	*N* = *45*		*N* = *73*	*N* = *54*	*N* = *19*	
Age	52.0 [45.0;59.2]	51.0 [45.0;56.0]	53.0 [45.0;68.0]	0.102	53.0 [47.0;62.0]	53.0 [47.0;61.0]	52.0 [46.0;65.5]	0.796
Symptom:				0.417				0.493
None	119 (70.8%)	84 (68.3%)	35 (77.8%)		49 (67.1%)	34 (63.0%)	15 (78.9%)	
Nipple discharge	15 (8.93%)	13 (10.6%)	2 (4.44%)		7 (9.59%)	6 (11.1%)	1 (5.26%)	
Palpable lump	34 (20.2%)	26 (21.1%)	8 (17.8%)		17 (23.3%)	14 (25.9%)	3 (15.8%)	
Lesion size	23.0 [15.0;31.2]	23.0 [16.0;31.5]	22.0 [12.0;29.0]	0.186	26.0 [19.0;35.0]	28.0 [19.2;35.8]	21.0 [15.5;30.0]	0.079
USMorphology:				0.005				0.020
Mass	73 (43.5%)	45 (36.6%)	28 (62.2%)		39 (53.4%)	24 (44.4%)	15 (78.9%)	
Nonmass lesion	95 (56.5%)	78 (63.4%)	17 (37.8%)		34 (46.6%)	30 (55.6%)	4 (21.1%)	
MGMorphology:				< 0.001				< 0.001
Noncalcified	59 (35.1%)	33 (26.8%)	26 (57.8%)		27 (37.0%)	13 (24.1%)	14 (73.7%)	
Calcified	109 (64.9%)	90 (73.2%)	19 (42.2%)		46 (63.0%)	41 (75.9%)	5 (26.3%)	
US-BI RADS:				0.003				0.002
3	23 (13.7%)	10 (8.13%)	13 (28.9%)		13(17.8%)	5 (9.26%)	8(42.1%)	
4A	26 (15.5%)	19 (15.4%)	7 (15.6%)		11 (15.1%)	8 (14.8%)	3 (15.8%)	
4B	47 (28.0%)	33 (26.8%)	14 (31.1%)		18 (24.7%)	12 (22.2%)	6 (31.6%)	
4C	54 (32.1%)	44 (35.8%)	10 (22.2%)		23 (31.5%)	22 (40.7%)	1 (5.26%)	
5	18 (10.7%)	17 (13.8%)	1 (2.22%)		8 (11.0%)	7 (13.0%)	1 (5.26%)	
MG-BI RADS:				0.001				0.001
2	9 (5.36%)	3 (2.44%)	6 (13.3%)		6 (8.22%)	2 (3.70%)	4 (21.1%)	
3	33 (19.6%)	17 (13.8%)	16 (35.6%)		11 (15.1%)	5 (9.26%)	6 (26.3%)	
4A	32 (19.0%)	25 (20.3%)	7 (15.6%)		18 (24.7%)	13 (24.1%)	5 (26.3%)	
4B	61 (36.3%)	49 (39.8%)	12 (26.7%)		22 (30.1%)	20 (37.0%)	2 (10.5%)	
4C	30 (17.9%)	26 (21.1%)	4 (8.89%)		15 (20.5%)	14 (25.9%)	1 (5.26%)	
5	3 (1.79%)	3 (2.44%)	0 (0.00%)		1 (1.37%)	0 (0.00%)	1 (5.26%)	
ER:				0.004				0.027
Negative	39 (23.2%)	36 (29.3%)	3 (6.67%)		20 (27.4%)	19 (35.2%)	1 (5.26%)	
Positive	129 (76.8%)	87 (70.7%)	42 (93.3%)		53 (72.6%)	35 (64.8%)	18 (94.7%)	
PR:				0.005				0.106
Negative	38 (22.6%)	35 (28.5%)	3 (6.67%)		20 (27.4%)	18 (33.3%)	2 (10.5%)	
Positive	130 (77.4%)	88 (71.5%)	42 (93.3%)		53 (72.6%)	36 (66.7%)	17 (89.5%)	
HER2:				< 0.001				0.003
NA	13 (7.74%)	10 (8.13%)	3 (6.67%)		9 (12.3%)	7 (13.0%)	2 (10.5%)	
Negative	115 (68.5%)	74 (60.2%)	41 (91.1%)		45 (61.6%)	28 (51.9%)	17 (89.5%)	
Positive	40 (23.8%)	39 (31.7%)	1 (2.22%)		19 (26.0%)	19 (35.2%)	0 (0.00%)	
KI67:				0.001				0.004
Negative	132 (78.6%)	88 (71.5%)	44 (97.8%)		56 (76.7%)	37 (68.5%)	19 (100%)	
Positive	36 (21.4%)	35 (28.5%)	1 (2.22%)		17 (23.3%)	17 (31.5%)	0 (0.00%)	

DCIS, ductal carcinoma in situ; ER, estrogen receptor; PR, progesterone receptor; HER2, human epidermal growth factor receptor 2; MG, mammography; US, ultrasound; BI-RADS, Breast Imaging Reporting and Data System.

### Feature selection

From the regions of interest (ROIs) in DCIS US and MG images, we extracted a total of 105 radiomic features from each imaging modality, resulting in 210 features overall. These features included 14 shape features, 18 first-order features (e.g., intensity histogram features), and 73 higher-order features (e.g., texture features) per modality. The complete list of radiomic features is provided in Supplementary Table S3. After evaluating the reproducibility of the 210 extracted features using ICC, we retained features with ICC > 0.8 for subsequent analysis. This process resulted in 162 robust features: 84 from US images and 78 from MG images. Using LASSO regression, three different models were constructed: the US radiomic model, the MG radiomic model, and the US + MG radiomic model. As shown in Supplementary Table S4, the US + MG radiomic model demonstrated the best diagnostic performance, achieving an AUC of 0.83 [0.81–0.86] in the training cohort and 0.81 [0.77–0.84] in the validation cohort. The US + MG radiomic model includes 4 predictive features with non-zero coefficients: two from US images (Maximum Probability and Autocorrelation) and two from MG images (Skewness and Cluster Shade). The radiomic score was then computed by utilizing these features and their corresponding coefficients (Eq. 1). The feature selection process is depicted in Supplementary Fig. S1 and S2.

1 $$\begin{aligned} {\text{Radiomicscore}} & {\mkern 1mu} = {\mkern 1mu} 1.32{\mkern 1mu} + {\mkern 1mu} 0.35{\mkern 1mu} \times {\mkern 1mu} {\text{Autocorrelation}}{\mkern 1mu} + {\mkern 1mu} 0.05{\mkern 1mu} \times {\mkern 1mu} {\text{Skewness}} \\ & \;\; - {\mkern 1mu} 0.03{\mkern 1mu} \times {\mkern 1mu} {\text{MaximumProbability}}{\mkern 1mu} + {\mkern 1mu} 0.18{\mkern 1mu} \times {\mkern 1mu} {\text{ClusterShade}} \\ \end{aligned}$$

### Performance comparison between clinical and radiomic models

We trained two models by sequentially incorporating clinical and Radiomic scores: (i) clinical features, including age, symptoms, and five imaging features; and (ii) Radiomic scores. Upon completion of training, the models were frozen, and their optimal hyperparameters were determined and tested on the validation cohort. The optimal hyperparameter combinations are provided in Supplementary Table S5. All potential predictive variables, including age, symptoms, and nodule size, were included in the model despite not showing significant differences between groups, as they might be beneficial for model training. Collinearity diagnostics were conducted before model training, revealing VIF values below 2 for all features and correlation coefficients below 0.7 in the correlation matrix heatmap (Supplementary Table S6, Fig. S3). In the training cohort, the clinical model demonstrated moderate diagnostic performance across individual classifiers, with AUCs ranging from 0.75 to 0.78, and an ensemble classifier achieving the best AUC of 0.87 (95% CI 0.81–0.93). Similarly, in the validation cohort, the clinical model’s ensemble classifier outperformed individual classifiers, achieving an AUC of 0.86 (95% CI 0.76–0.97). The Radiomic ensemble model exhibited superior diagnostic performance compared to the clinical model. In the training cohort, individual classifiers within the Radiomic model achieved AUCs ranging from 0.80 to 0.85, with the ensemble classifier attaining an AUC of 0.94 (95% CI 0.91–0.98). In the validation cohort, the Radiomic model achieved AUCs ranging from 0.78 to 0.84 across individual classifiers, with the ensemble classifier achieving the best AUC of 0.92 (95% CI 0.84–0.96). The comprehensive performance comparison of both models is detailed in Table 3.

**Table 3:** Diagnostic performance of models for low nuclear grade DCIS.

Dataset	Model	AUC (95% CI)	Accuracy	Sensitivity	Specificity	NPV	PPV
Training cohort	Clinical model (Ranger)	0.78 (0.72–0.84)	0.70	0.72	0.65	0.88	0.58
	Clinical model (Elastic net)	0.75 (0.68–0.82)	0.68	0.68	0.68	0.85	0.55
	Clinical model (GLMBoost)	0.76 (0.69–0.83)	0.69	0.70	0.66	0.86	0.57
	Clinical model (Ensemble)	0.87 (0.81–0.93)	0.82	0.84	0.78	0.91	0.64
	Radiomic model (Ranger)	0.85 (0.80–0.91)	0.78	0.80	0.70	0.91	0.65
	Radiomic model (Elastic net)	0.80 (0.72–0.87)	0.72	0.68	0.82	0.83	0.55
	Radiomic model (GLMBoost)	0.82 (0.75–0.88)	0.75	0.75	0.74	0.87	0.60
	Radiomic ensemble model	0.94 (0.91–0.98)	0.89	0.88	0.91	0.96	0.73
Validation cohort	Clinical model (Ranger)	0.75 (0.68–0.82)	0.68	0.70	0.64	0.86	0.57
	Clinical model (Elastic net)	0.72 (0.65–0.80)	0.65	0.66	0.67	0.83	0.54
	Clinical model (GLMBoost)	0.73 (0.66–0.80)	0.66	0.68	0.65	0.84	0.56
	Clinical model (Ensemble)	0.86 (0.76–0.97)	0.85	0.85	0.84	0.94	0.67
	Radiomic model (Ranger)	0.84 (0.77–0.91)	0.77	0.78	0.69	0.90	0.63
	Radiomic model (Elastic net)	0.78 (0.69–0.86)	0.70	0.65	0.80	0.81	0.50
	Radiomic model (GLMBoost)	0.80 (0.73–0.87)	0.72	0.72	0.73	0.85	0.55
	Radiomic ensemble model	0.92 (0.84–0.96)	0.88	0.89	0.84	0.94	0.73

DCIS, ductal carcinoma in situ; AUC, area under the curve; CI, confidence interval; NPV, negative predictive value; PPV, positive predictive value; GLMBoost, Generalized Linear Models with Boosting.

### The radiomic ensemble model’s gradual increase in predictive value

**Fig. 3:**
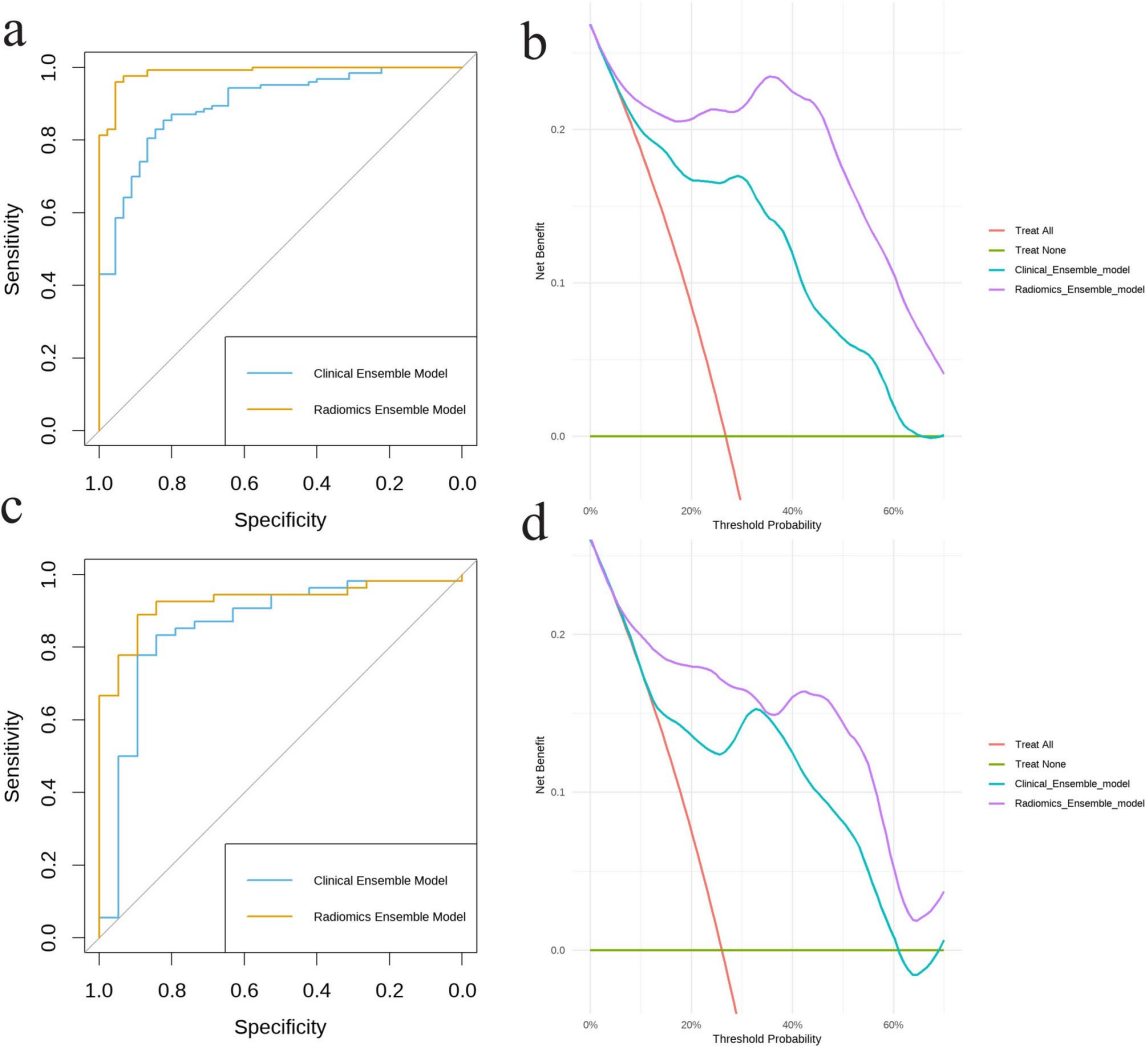
Performance and decision curve analysis of the clinical and Radiomic ensemble models. (**a**, **b**) ROC curves for the training and validation cohorts. (**c**, **d**) Decision curve analysis for the training and validation cohorts.

We assessed the additional predictive capability of Radiomic scores for LNG DCIS by utilizing IDI and NRI. The inclusion of Radiomic characteristics in the clinical model led to an increase in the Integrated Discrimination Improvement (IDI) of 0.12 (95% CI 0.10–0.15, *p* < 0.001) in the training group and 0.10 (95% CI 0.07–0.13, *p* < 0.001) in the validation group. In the training group, the NRI was 1.32 (95% CI 1.07–1.52, *p* < 0.001), while in the validation group, it was 1.30 (95% CI 0.96–1.60, *p* < 0.001). The ROC curves in Fig. 3a and b further demonstrate the superior discriminatory performance of the Radiomic ensemble model compared to the clinical model, with consistently higher AUC values across both training and validation cohorts. Additionally, DCA in both cohorts demonstrated that the Radiomic ensemble model provided a higher net benefit across a wide range of threshold probabilities (10%-70%) compared to the clinical model (Fig. 3c, d).

### Predictive potential for DFS

**Fig. 4:**
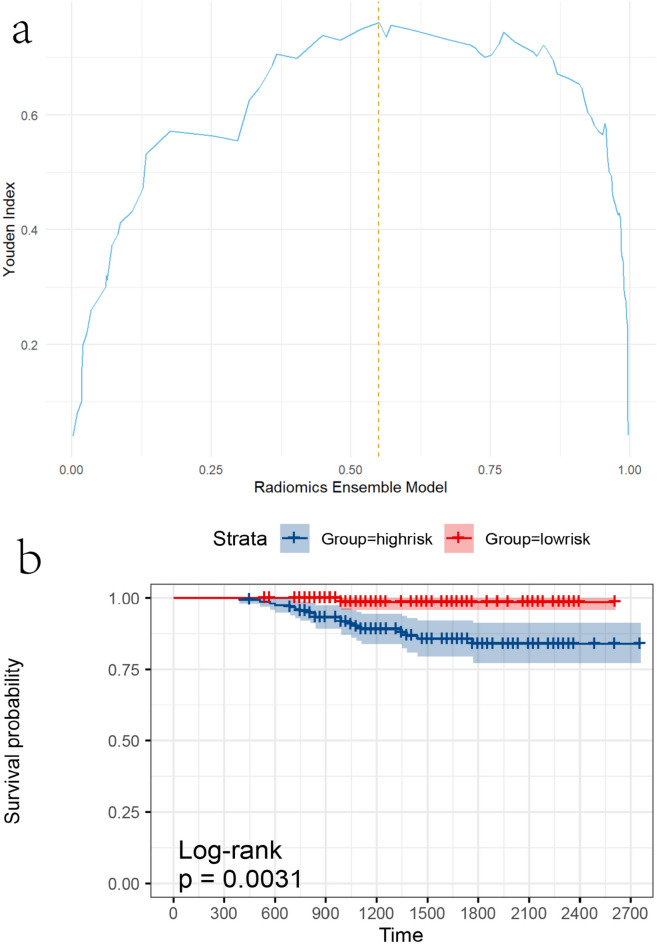
Radiomic ensemble model risk stratification and DFS analysis. (**a**) Stratification of high- and low-risk groups based on Youden’s index cut-off point. (**b**) Kaplan–Meier plot illustrating the correlation between the Radiomic ensemble model’s predictions and disease-free survival (DFS).

Using a threshold of 0.55, the Radiomic ensemble model categorized individuals into high-risk (prediction > 0.55) and low-risk (prediction ≤ 0.55) groups (Fig. 4a). Follow-up data were available for all included patients, with a median follow-up duration of 1350 days (interquartile range: 990–1980 days). During this period, 20 DFS-related events (8.3%) were observed. Kaplan–Meier analysis demonstrated a significant association between risk groups and DFS outcomes, with the Radiomic ensemble model effectively stratifying patients into high-risk and low-risk categories (log-rank test, *p* < 0.0031; Fig. 4b). These findings underscore the potential utility of the model in guiding personalized treatment strategies and optimizing clinical decision-making.

### Model interpretation and feature importance

**Fig. 5:**
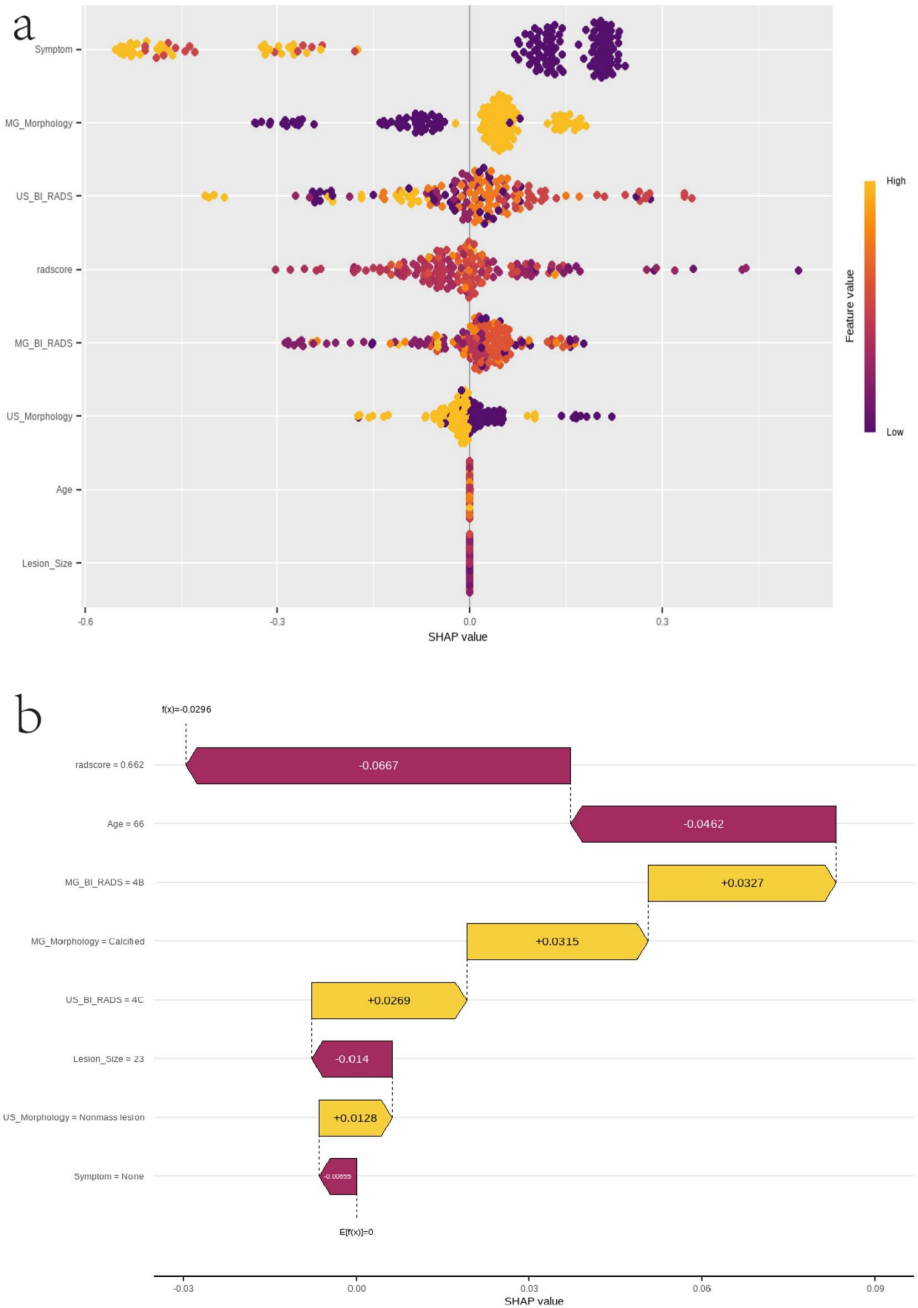
SHAP values interpretation and feature importance. (**a**) SHAP summary plot illustrating the influence of each feature on model predictions, emphasizing the integration of radiomic, clinical, and imaging features. (**b**) Example case of LNG DCIS confirmed through surgical pathology, with a SHAP waterfall plot showing individual feature contributions to the model’s prediction.

SHAP values were used to explain the model and evaluate the significance of features. SHAP values quantify each feature’s contribution to the model’s predictions. A SHAP summary plot was created to provide a comprehensive overview of how different features influenced the model’s predictions (Fig. 5a). The plot highlights the complementary value of integrating radiomic, clinical, and imaging features, demonstrating that their combination enhances the model’s performance in predicting LNG DCIS. Additionally, Fig. 5b illustrates a specific case of LNG DCIS confirmed through surgical pathology. The SHAP waterfall plot visualizes the contributions of individual features to this prediction, offering a transparent and interpretable perspective on the model’s decision-making process. These results underscore the importance of leveraging multimodal data for robust and clinically meaningful predictions of LNG DCIS.

## Discussion

In this study, we developed an integrated Radiomic ensemble model utilizing US and MG for preoperative prediction of LNG DCIS. By employing three algorithms and five random seeds, we constructed an ensemble model incorporating clinical features, US images, MG images, and Radiomic scores, significantly enhancing the preoperative prediction of LNG DCIS. Notably, the ensemble model consistently outperformed individual classifiers across both the clinical and Radiomic models, achieving the highest AUC in the validation cohort. Specifically, the Radiomic ensemble model achieved an AUC of 0.92 in the validation cohort, compared to 0.78–0.84 for individual classifiers within the same model, further demonstrating the added value of integrating Radiomic features. Furthermore, we demonstrated that the Radiomic ensemble model significantly improved diagnostic performance compared to the clinical model, highlighting its robustness and potential utility in clinical decision-making. Lastly, the ensemble model effectively stratified DCIS patients by DFS risk, underscoring its potential to guide personalized treatment strategies and improve patient outcomes.

A recent study^17^ reported findings on the histological grading of DCIS, indicating that if a mass is observed on US, no microcalcification is present on MG, and no comedo necrosis is detected on biopsy, the final pathology is likely to be LNG DCIS, with an AUC of 0.97. However, this study focused on identifying factors that could lead to the upgrading of LNG DCIS diagnosed by preoperative imaging and biopsy upon surgery. In reality, the nuclear grade of DCIS can be identified using preoperative core needle biopsy or vacuum-assisted biopsy. The presence of comedo necrosis in the preoperative biopsy pathology suggests that the lesion is not likely to be LNG DCIS, rendering further diagnostic information unnecessary. In contrast, our study aimed to utilize readily available clinical imaging data and derived Radiomic features for the non-invasive preoperative prediction of LNG DCIS. Radiomic has demonstrated tremendous potential in various challenging clinical tasks^33–35^, and our results support this assertion. By incorporating Radiomic scores into the clinical model, the IDI and NRI significantly improved (*p* < 0.001), resulting in substantial clinical benefit. Given the widespread use of US and MG, our approach may be more easily replicated in other healthcare settings, such as primary care or remote areas, where preoperative biopsy equipment and expertise are limited^36–38^.

Our study also significantly differs from previous reports. Firstly, unlike prior studies^28,39^ that focused solely on single-modality Radiomic analysis (MRI or ultrasound), our study leveraged the combined use of dual-modality Radiomic features with ensemble machine learning algorithms, resulting in superior predictive performance (AUC 0.92 vs. 0.61–0.77). Secondly, we performed LASSO regression on US-only, MG-only, and US + MG combined radiomic features. The US + MG model demonstrated the best diagnostic performance, emphasizing the complementary role of both imaging modalities in predicting LNG DCIS. This further supports the value of combining multimodal radiomic features for improved diagnostic accuracy. A recent publication^40^ proposed a multimodal deep learning tri-classification model based on ultrasound for identifying the surgical pathology of DCIS. Although this study also utilized multiple imaging data, its primary focus was on identifying the entire DCIS spectrum rather than specifically LNG DCIS. Moreover, deep learning algorithms are frequently condemned for being ‘black box’ models, lacking transparency and interpretability.

Our study aimed to enhance interpretability by including Radiomic features with clearly defined formulas and biological significance, offering transparency and interpretability in clinical applications. Consistent with findings in Boehm et al.^41^ and Cozzi et al.^42^, texture-based radiomic features, such as Maximum Probability, Autocorrelation, Skewness, and Cluster Shade, played pivotal roles in our model. These features capture critical characteristics of tissue structure and texture, providing valuable insights into tumor biology. Specifically, Autocorrelation and Cluster Shade reflect the uniformity and regularity of tissue structures, aligning with the known characteristics of LNG DCIS as less aggressive and more homogeneous. Maximum Probability and Skewness, on the other hand, quantify the concentration and symmetry of pixel intensity distributions, correlating with more symmetrical and organized cell patterns. These findings underscore the potential of radiomic features in characterizing tumor heterogeneity and linking imaging data to biological behavior. Compared to prior studies^43^ that utilized features like entropy to describe heterogeneity, our study highlights the value of texture-based features that are robust, reproducible, and directly interpretable. This distinction reinforces the clinical utility of our model and aligns with recent efforts to integrate Radiomic into diagnostic workflows^44,45^. Furthermore, by leveraging SHAP analysis, we provided a transparent framework for understanding the contribution of each feature to the model’s predictions. This approach not only ensures interpretability but also bridges the gap between complex computational models and clinical practice. The SHAP-derived insights empower clinicians to validate the model’s decisions and integrate them into diagnostic pathways, improving confidence in the model’s outputs. Finally, our model demonstrates practical value in resource-limited settings. By integrating radiomic features with clinical data, the model serves as an efficient and cost-effective tool to complement preoperative biopsy, reducing reliance on invasive procedures while enhancing diagnostic accuracy. These advantages are particularly relevant for primary care and remote areas, where access to advanced diagnostic tools is limited^46^^.^ Future studies involving large, multicenter datasets will further validate these findings and explore the broader applicability of the model in diverse clinical settings.

This study also confirmed previous observations that LNG DCIS is associated with mass-like lesions, whereas HNG DCIS is more commonly associated with non-mass lesions and microcalcifications^47^. HNG DCIS more frequently exhibits malignant features on US and MG^48^, potentially explaining the higher BI-RADS scores for intermediate to high nuclear grade DCIS compared to LNG DCIS. In our study, microcalcifications were present in 37.5% of LNG DCIS cases and 62.5% of intermediate to high nuclear grade DCIS cases. Moreover, increased BI-RADS scores were associated with more serious categorizations, as LNG DCIS was typically categorized as 4B or lower, while intermediate to high nuclear grade DCIS was more commonly classified as 4C or 5. Prior research indicates that LNG DCIS tends to have higher rates of ER and PR positivity and lower rates of comedo necrosis^48,49^, whereas HNG DCIS is more frequently positive for Ki-67 and HER2^17,23^. Our findings align with prior research, providing additional evidence for the link between these clinicopathological characteristics and the diversity of DCIS, while also elucidating the enhanced model accuracy achieved through the integration of multimodal information from both ultrasound and mammography. Importantly, our model effectively stratified DCIS patients by recurrence risk (*P* < 0.05)^50^, identifying patients at higher risk of recurrence, thereby optimizing patient management strategies and reducing unnecessary invasive examinations and treatments.

There are various constraints to this study. Firstly, the sample size of 241 cases is relatively modest, which may limit the robustness and generalizability of the model. To mitigate this limitation, we employed rigorous validation techniques, including five-fold cross-validation, random seed experiments, and the use of an independent validation cohort, to ensure consistent and reliable performance. Secondly, although we included various imaging and clinical features, some potential confounding factors, such as preoperative biopsy information, were not accounted for. Thirdly, the model was trained and validated using retrospective data from a single institution, which may introduce center-specific effects and selection bias. The inclusion and exclusion criteria were designed to capture a representative spectrum of DCIS cases encountered in clinical practice, but we acknowledge that retrospective studies are inherently prone to selection bias. Future studies should include larger, multicenter prospective datasets to enhance the generalizability and stability of the model. Additionally, a potential challenge is the labor-intensive nature of manual ROI segmentation. Advanced deep learning models are quickly being developed for automated segmentation in different types of cancer, offering the potential to greatly decrease segmentation time and enhance effectiveness in the coming years.

In conclusion, we successfully developed a Radiomic ensemble model utilizing US and MG multimodal features for the preoperative prediction of LNG DCIS. The internal validation cohort showed that the model had outstanding predictive accuracy and successfully categorized DCIS patients based on DFS risk. Patients with probabilities greater than or equal to the threshold should receive more attention due to their increased recurrence risk. Future studies should validate the model’s efficacy in multicenter settings and explore automatic segmentation techniques to enhance efficiency.

## Supplementary Information

Supplementary Information.

## Data Availability

The data sets utilized and/or examined in the present research can be obtained from the corresponding author upon request.
