# Supplementary material for: Integrating radiomics into predictive models for low nuclear grade DCIS using machine learning
Source: Sci Rep. 2025 Mar 3;15:7505. doi: 10.1038/s41598-025-92080-y (PMC11876686; doi:10.1038/s41598-025-92080-y)
Supplement: Supplementary file 1 — Supplementary Information. [file 41598_2025_92080_MOESM1_ESM.pdf]

Supplementary Information

A. Supplementary figures

Fig. S1 Feature Selection Process Using ICC

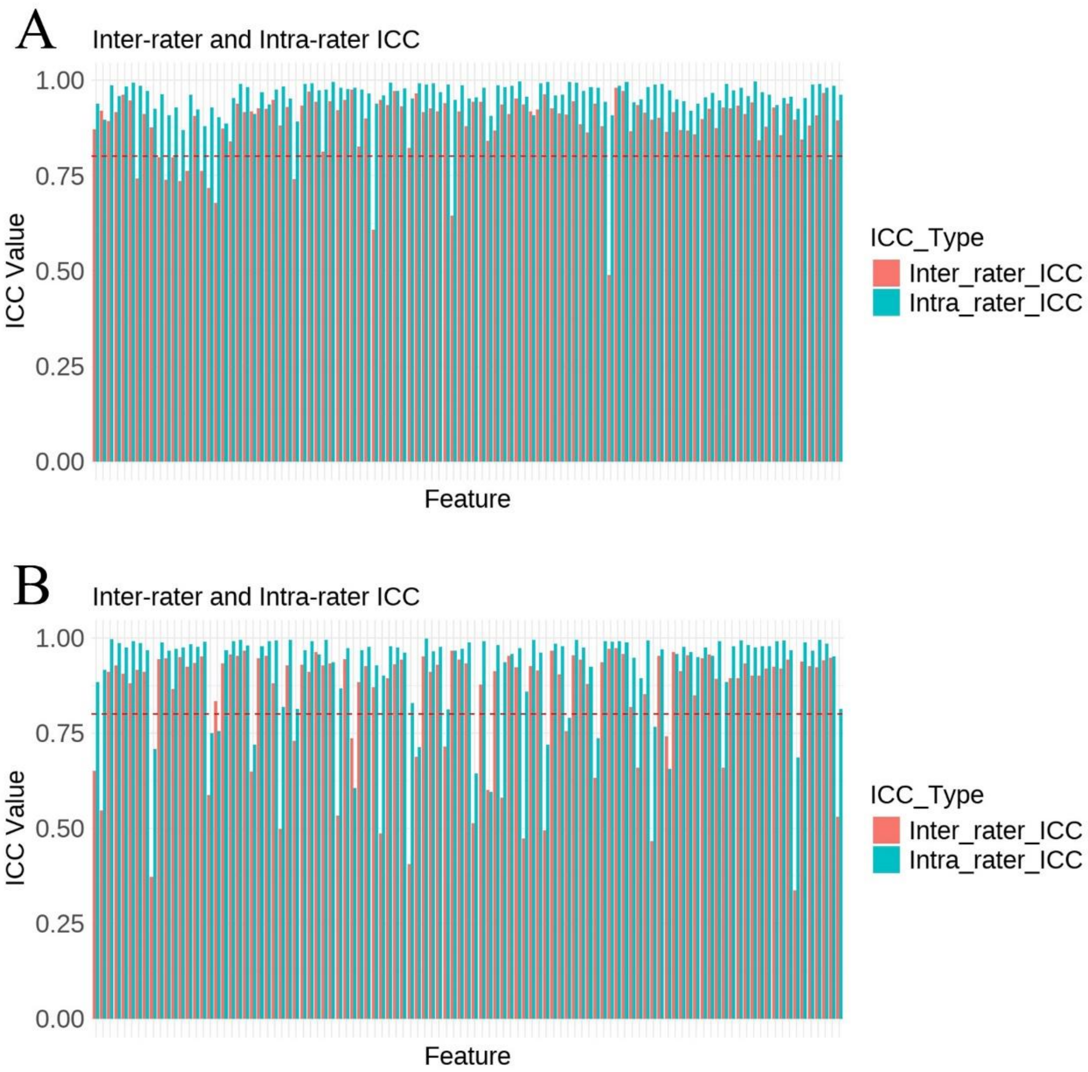

Fig. S1 Inter-rater and intra-rater consistency checks (ICC) results.(A) ICC values for Ultrasound (US) radiomics features.(B) ICC values for Mammography (MG) radiomics features.

Fig. S2 Feature Selection Process Using LASSO

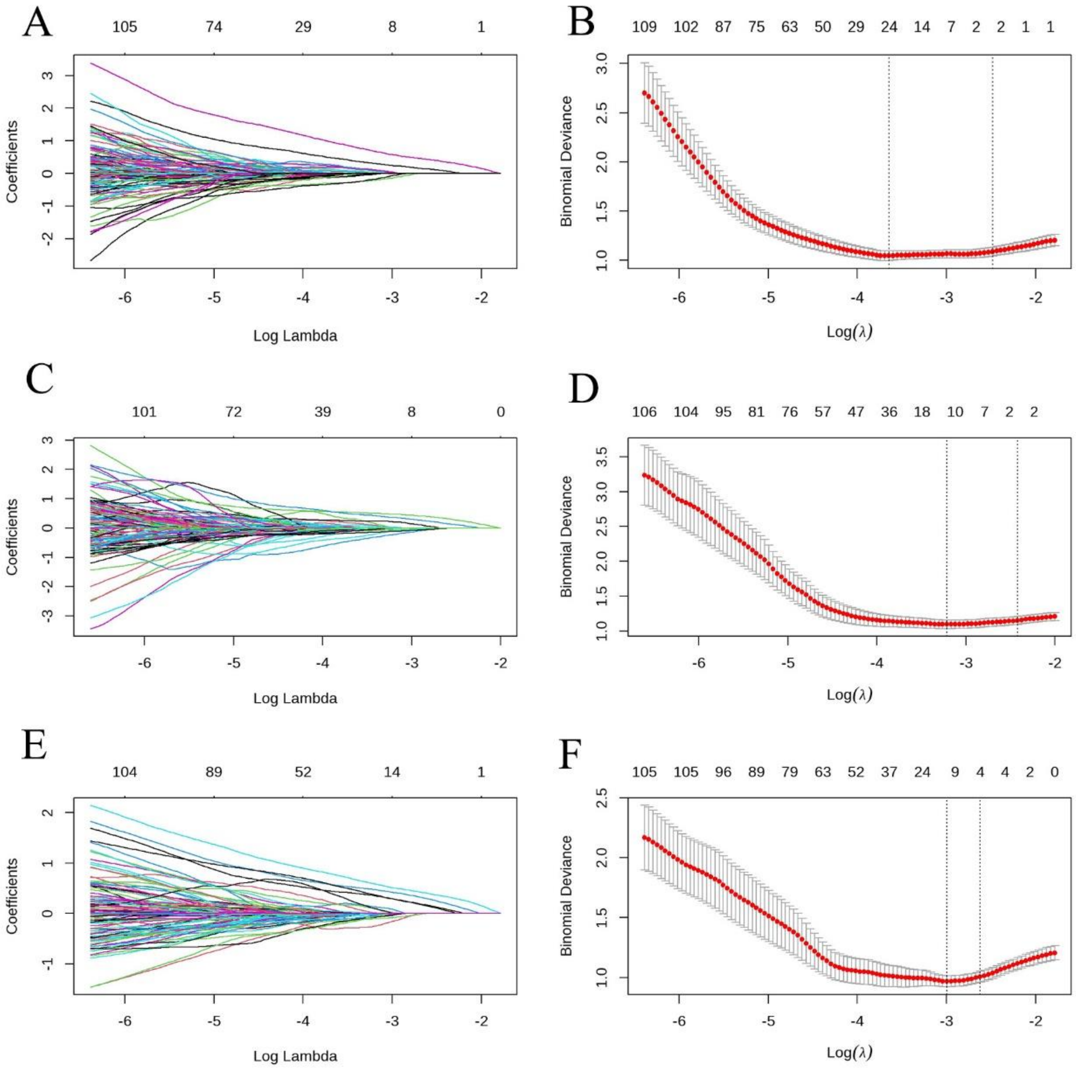

Fig. S2 (A&B) Use LASSO to select US-Radiomics features. (C&D) Use LASSO to select MG-Radiomics. (E&F) Use LASSO to select US+MG Radiomics features.

Fig. S3 Correlation matrix of clinical and radiomics features

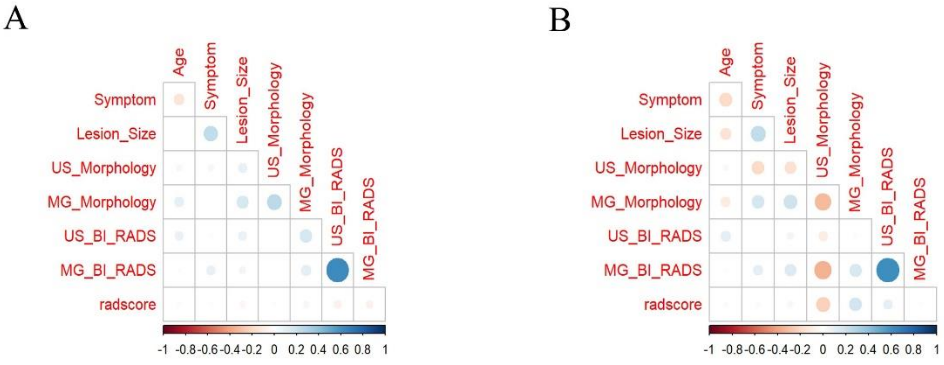

Fig. S3 (A) Correlation matrix plot (training cohort), (B) Correlation matrix plot (validation cohort).

B. Supplementary tables

Table S1. Overview of All Features Used in the Study

Category	Feature Name	Description	Data Type/Scale
Clinical	Age	Patient age at diagnosis (years)	Continuous (years)
Clinical	Symptoms	Clinical presentation (e.g., palpable lump, nipple discharge)	Categorical (Yes/No)
Clinical	ER Status	Estrogen receptor status	Categorical (Positive/Negative)
Clinical	PR Status	Progesterone receptor status	Categorical (Positive/Negative)
Clinical	HER2 Status	Human Epidermal growth factor Receptor 2 status	Categorical (Positive/Negative)
Clinical	Ki-67	Proliferation marker (Ki-67)	Categorical (≥14% or <14%)
Imaging (US)	Lesion Size	Largest diameter of the lesion (mm)	Continuous (mm)
Imaging (US)	US Morphology	Mass or non-mass lesion	Categorical (Mass/Non-mass)
Imaging (US)	US-BI RADS	Ultrasound BI-RADS category	Ordinal (e.g., 4A, 4B, 4C, 5)
Imaging (MG)	MG Morphology	Presence of microcalcifications	Categorical (Yes/No)
Imaging (MG)	MG-BI-RADS	Mammography BI-RADS category	Ordinal (e.g., 4A, 4B, 4C, 5)
Radiomics (US)	Autocorrelation	Texture feature quantifying repeated intensity patterns in the ROI	Continuous
Radiomics (US)	Maximum Probability	Texture feature representing the highest occurrence probability of certain intensity pairs	Continuous
Radiomics (MG)	Skewness	Intensity histogram feature measuring the asymmetry of intensity distribution	Continuous
Radiomics (MG)	Cluster Shade	Texture feature indicating skewness and uniformity in the GLCM	Continuous

MG, mammography; US, ultrasound; BI-RADS, Breast Imaging Reporting and Data System; GLCM, gray level co-occurrence matrix; ER Status, Estrogen receptor status; PR Status, Progesterone receptor status.

Table S2 Hyperparameter ranges used in the cross-validation model optimisation.

Algorithm	Hyperparameter	Range
Elastic net	L1 ratio	0-1 (Automatic)
Glmboost	mstop	50-300 (steps of 1)
Ranger	num.trees	100-2000 (steps of 1)
Ranger	min.node.size	1-100 (steps of 1)
Ranger	sample.fraction	0.1-1.0 (Automatic)
Ranger	mtry	1-8 (steps of 1)

Glmboost, Generalized Linear Models with Boosting.

Table S3 The summary of the 105 radiomic features.

Feature subgroups	Number	Representative 3-4 feature details
Shape	12	Voxel Volume, Maximum 3D Diameter, Surface Area
First order	18	Interquartile Range, Skewness, Uniformity
GLCM	24	Joint Average, Sum Average, Joint Entropy
GLDM	14	Gray Level Variance, High Gray Level Emphasis, Dependence Entropy
GLRLM	16	Short Run Low Gray Level Emphasis, Run Variance, Gray Level Variance, Low Gray Level Run Emphasis
GLSZM	16	Zone Variance, Size Zone Non-Uniformity, Zone Percentage, Small Area Emphasis
NGTDM	5	Coarseness, Complexity, Strength, Contrast
Total	105	

GLDM, gray level dependence matrix; GLCM, gray level co-occurrence matrix; GLRLM, gray-level run-length matrix; GLSZM, gray-level size zone matrix; NGTDM, neighborhood gray-tone difference matrix.

Table S4. Performance Comparison of Three Models Based on LASSO regression

**Model**	**Training set (N = 168)**			**validation set (N = 73)**		
	sensitivity	specificity	AUC (95% CI)	sensitivity	specificity	AUC (95% CI)
US-Radiomics	0.71	0.69	0.74 [0.71-0.83]	0.67	0.73	0.73 [0.68-0.81]
MG-Radiomics	0.68	0.66	0.70 [0.67-0.82]	0.69	0.64	0.71 [0.67-0.81]
US+MG Radiomics	0.77	0.74	0.83 [0.81-0.86]	0.72	0.76	0.81 [0.77-0.84]

LASSO, Least Absolute Shrinkage and Selection Operator; MG, mammography; US, ultrasound; AUC, Area under the receiver operating characteristic curve; 95%CI, 95% confidence intervals.

Table S5 Optimised hyperparameters used in each of the algorithms and seeds.

Algorithm	Hyperparameter	Seed1	Seed2	Seed3	Seed4	Seed5
Elastic net	L1 ratio	0.4680161	0.7501852	0.2384159	0.8477173	0.08694806
Glmboost	mstop	238	74	111	76	103
Ranger	num.trees	1379	804	1461	1170	1235
Ranger	min.node.size	13	21	4	5	20
Ranger	sample.fraction	0.678381	0.6519635	0.2356834	0.407339	0.8364323
Ranger	mtry	1	3	3	2	1

Glmboost, Generalized Linear Models with Boosting.

Table S6 Results of Variable Collinearity Analysis

Dataset	Variables	VIF
Training cohort	Age	1.031784
	Symptom	1.119513

	Lesion size	1.112410
	US morphology	1.076842
	MG morphology	1.132224
	US-BI RADS	1.757312
	MG-BI RADS	1.723362
	radscore	1.007966
Validation cohort	Age	1.060734
	Symptom	1.118716
	Lesion size	1.119932
	US morphology	1.326225
	MG morphology	1.179548
	US-BI RADS	1.652763
	MG-BI RADS	1.835659
	radscore	1.094548

MG, mammography; US, ultrasound; BI-RADS, Breast Imaging Reporting and Data System, VIF, Variance Inflation Factor.
